# Nematode Orthologs of Macrophage Migration Inhibitory Factor (MIF) as Modulators of the Host Immune Response and Potential Therapeutic Targets

**DOI:** 10.3390/pathogens11020258

**Published:** 2022-02-17

**Authors:** Justyna Karabowicz, Ewa Długosz, Piotr Bąska, Marcin Wiśniewski

**Affiliations:** 1 Division of Parasitology and Parasitic Diseases, Department of Preclinical Sciences, Institute of Veterinary Medicine, Warsaw University of Life Sciences—SGGW, 02-786 Warsaw, Poland; ewa_dlugosz@sggw.edu.pl (E.D.); marcin_wisniewski@sggw.edu.pl (M.W.); 2 Division of Pharmacology and Toxicology, Department of Preclinical Sciences, Institute of Veterinary Medicine, Warsaw University of Life Sciences—SGGW, 02-786 Warsaw, Poland; piotr_baska@sggw.edu.pl

**Keywords:** macrophage migration inhibitory factor, Nematoda, immunomodulation, orthologous proteins

## Abstract

One of the adaptations of nematodes, which allows long-term survival in the host, is the production of proteins with immunomodulatory properties. The parasites secrete numerous homologs of human immune mediators, such as macrophage migration inhibitory factor (MIF), which is a substantial regulator of the inflammatory immune response. Homologs of mammalian MIF have been recognized in many species of nematode parasites, but their role has not been fully understood. The application of molecular biology and genetic engineering methods, including the production of recombinant proteins, has enabled better characterization of their structure and properties. This review provides insight into the current state of knowledge on MIF homologs produced by nematodes, as well as their structure, enzymatic activity, tissue expression pattern, impact on the host immune system, and potential use in the treatment of parasitic, inflammatory, and autoimmune diseases.

## 1. Introduction

Parasitic nematodes modulate the immune response to ensure their prolonged survival in the host. The long lifespan of parasites provides ample evidence that they are extremely adept at evading the immune system, and it is clear that interference and modulation are among the first events to occur during infection [1,2,3]. Molecules released by nematodes modulating the host immune response include antioxidants, proteases, protease inhibitors, and orthologs of cytokines and their receptors [4,5]. A strategy of particular interest is mimicking host immune system molecules. Nematodes release a number of homologs of human immune components, such as TGF-β and macrophage migration inhibitory factor (MIF) [6].

Mammalian MIF (mMIF) is a proinflammatory cytokine with pleiotropic functions and a significant regulator of the inflammatory immune response. Homologs of mMIF have been recognized in numerous parasite species belonging to protozoa and helminths. The role of MIF orthologs from these organisms is not fully understood yet, although several reports indicate they play an important role in host immune response evasion and immunomodulation strategies. A better comprehension of the nature of these molecules and mechanisms via which they affect the immune reactivity will be critical in comprehending immune-mediated pathogenesis and developing effective therapies [7,8] against the intruders.

This article reviews the existing knowledge about MIF homologs produced by parasitic nematodes. We focus especially on their immunomodulatory properties, which may be used in the treatment of parasitic diseases, allergies, and autoimmune diseases.

## 2. MIF Structure and Function

**Figure 1:**
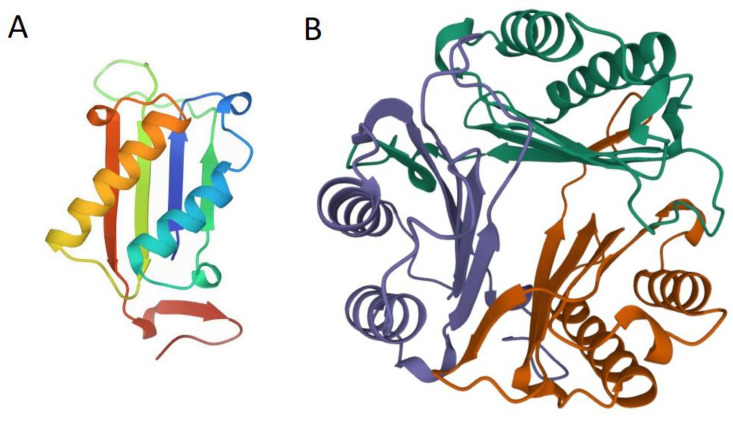
Human MIF monomer (**A**) and trimer (**B**) crystal structures retrieved from the Protein Data Bank (PDB 1MIF). Three subunits (green, violet, and orange) are shown (**B**). The α helices and β sheets are plotted.

MIF was discovered in 1966 and described as a T-cell-derived mediator with the specific property of inhibiting the random movement of macrophages [9]. It is a pleiotropic proinflammatory protein with numerous biological functions [10,11]. The MIF gene in humans is located on chromosome 22, composed of 114 amino acids with a mass of 12.5 kDa [12,13,14]. MIF has a homotrimeric structure, with each monomer containing a β–α–β motif, as shown in Figure 1 [15,16,17].

Its conformation is well conserved in eukaryotes: protozoans, animals (from invertebrates to mammals), and plants. mMIFs (rat, mouse, human, bovine) show high homology (~90%) [18,19]. This molecule, unlike most cytokines, has enzymatic activity as a phenylpyruvate tautomerase. The conserved C–X–X–C motif is associated with oxidoreductase activity, and the *N*-terminal proline (Pro1) acts as a catalytic base for tautomerase activity [20,21,22], which may be inhibited by MIF inhibitor (ISO-1) and other small-molecule inhibitors [23,24]. Due to its tautomerase activity, MIF catalyzes tautomerization of d-dopachrome to generate 5,6-dihydroxyindole-2-carboxylic acid [25]. It is still unknown whether MIF enzymatic activity holds a physiological role in mammals; perhaps it only reflects a residual property of these proteins originating from their ancestral position in invertebrate immunity. It was also proven that MIF tautomerase activity is not linked with its role as an inhibitor of monocyte chemotaxis and migration. Two murine MIF mutants in which the *N*-terminal proline was replaced with either a serine or a phenylalanine remained capable of inhibiting monocyte chemotaxis despite significantly reduced or no phenylpyruvate tautomerase activity [26].

MIF is produced by a variety of cell types including macrophages, monocytes [27], neutrophils [28], eosinophils [29], lymphocytes [30], endothelial cells [31], epithelial cells [32], and smooth muscle cells [33]. MIF, unlike most cytokines, is constitutively expressed and stored in preformed “intracellular pools” during homeostasis [34]. It can be immediately released from the cells under inflammatory and stress stimulation, and its secretion can be identified without de novo synthesis. Because MIF lacks an *N*-terminal secretory sequence, it is released from cells through unconventional ER/Golgi secretory pathways [35]. In humans, MIF expression varies due to polymorphism in the upstream promoter region of the gene. A changeable number of CATT nucleotide repeats exist in this region, with 5–8 such sequences determining alternative alleles. The number of CATT repeats corresponds with the constitutive and inducible expression of the mRNA and protein [36].

MIF may exert its biological effects on cells through various cell signaling pathways. MIF binds to its receptor CD74 (MHC class II invariant chain), followed by formation of a complex with CD44 and activation of ERK1/2 and PI3K/Akt pathways. This in turn increases macrophage survival through inhibition of p53 activity [37,38,39]. Additionally, formation of other receptor complexes, such as CD74/CD44, CD74/CXCR2, CD74/CXCR4, and CD74/CXCR4/CXCR7, has been described [40]. Generally, activation of these complexes leads to inhibition of apoptosis and autophagy and to stimulation of cell proliferation and migration. The molecular mechanisms underlying these effects have been thoroughly reviewed by Bilsborrow et al. [41] and Jankauskas et al. [40].

An opposite role of MIF in regulation of cell death process was reported in 2016 [42]. The study identified MIF as a PARP-1-dependent nuclease associated with apoptosis inducing factor (AIF). AIF is required for MIF recruitment to the cell nucleus, where MIF cleaves genomic DNA into large fragments. Therefore, MIF possesses nuclease activity that is critical for PARP-1-dependent DNA damage and cell death in the parthanatos pathway. Another study showed that MIF knockdown enabled neuronal protection against parthanatos under conditions of simulated in vivo oxidative stress after spinal cord injury (SCI) [43]. Inhibition of MIF nuclease activity is a possible treatment target in diseases induced by PARP-1 overactivation [42,43].

MIF stimulates the expression of various cytokines, e.g., TNF-α, IL-1β, IL-6, IL-8, and IL-12, induces Toll-like receptor (TLR) 4 expression and release of nitric oxide, stimulates production of matrix metalloproteinases, cyclooxygenase 2, and prostaglandin E2, and inhibits the anti-inflammatory and immunosuppressive effects of glucocorticoids [11,18,44,45]. In the context of the mammalian immune system, MIF is a pluripotent and pleiotropic cytokine that plays critical roles in inflammatory and immune responses and in tumorigenesis. The most significant of MIF functions are its capacity to recruit cells of both innate and acquired immunity to the site of inflammation, and modulation of inflammatory activator (e.g., COX-2 nitric oxide, PGE2, and TLR4) expression along with the recruitment of inflammatory cells [10,46]. It also enables macrophage adherence, phagocytosis, and transendothelial migration, and it activates and enhances the release of proinflammatory cytokines via macrophages triggering a strong inflammatory response [47,48].

The proinflammatory properties of MIF make it a critical mediator of immune response against a wide range of pathogens, e.g., parasites [2]. Moreover, the detrimental role of this cytokine in various inflammatory and autoimmune conditions has been described in many studies. In humans, increased MIF expression has been linked to pathogenesis in several inflammatory conditions including cystic fibrosis, atherosclerosis, asthma, nephrotic syndrome, inflammatory bowel disease (IBD), multiple sclerosis, rheumatoid arthritis, and systemic lupus erythematosus [11,13,41,49,50,51,52,53].

## 3. MIF Homologs (nMIFs) in Parasitic Nematodes

Given that MIF is an evolutionarily old molecule, it is not surprising that similar genes possibly related to the mMIF superfamily (i.e., MIF and its d-dopachrome tautomerase (d-DT paralog) have been found in various prokaryotes and eukaryotes (e.g., plants, vertebrates such as fish, amphibians, birds, and mammals, and invertebrates such as protozoa, nematodes, mollusks, and arthropods) [19,54]. According to Michelet et al. [55], all known nematode species with publicly available genomic data contain MIF genes, except for the cyst nematodes *Globodera pallida* and *G. rostochiensi*. In the free-living nematode *Caenorhabditis elegans*, the MIF gene family contains four separate genes and the four corresponding proteins (*Ce*-MIF-1, -2, -3 and -4) with 15–32% amino-acid sequence identity to each other. The identity to human MIF (hMIF) is 22–35% [8,56].

**Figure 2:**
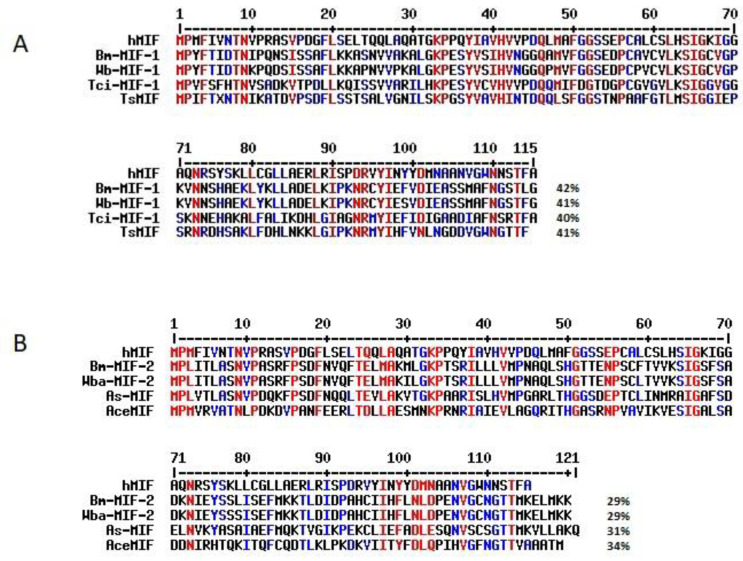
Alignment of selected nematode MIF-1 (**A**), MIF-2 (**B**), and hMIF protein sequences performed using MultiAlin tool [74]. Parasite MIF protein accession numbers are shown in Table 1; hMIF accession number: CAG30406.1. The protein identity (%) to hMIF calculated using BLASTp is indicated.

MIF homologs have been recognized in parasitic helminths belonging to the four major clades of the phylum Nematoda (reviewed by Vermeire et al. [8]). Among parasitic nematodes, MIF cDNA sequences have been reported in over 20 species (Table 1). Two different types of MIF homologs have been identified in several nematode species based on homology to *C. elegans* MIFs (*Ce*-MIF-1 and *Ce*-MIF-2) [8]. The homologs of *Ce*-MIF-1 have a higher level of amino-acid similarity to mMIFs than *Ce*-MIF-2 homologous proteins [8,56,57]. Some authors classified *Ce*-MIF-2 corresponding molecules from *B. malayi* and *O. volvulus* as d-DT homologs [58,59], which would explain the lower degree of similarity to their mammalian counterparts. Figure 2 shows an alignment of selected nematode MIF sequences with hMIF.

## 4. nMIFs: Structure, Function, Activity, and Expression

The tertiary structures of most nMIFs show a high level of similarity, despite limited homology to the amino-acid sequence. Similarly to hMIF, its nematode homologs form a homotrimeric molecule, which is essential for the protein’s catalytic activity [8]. The possibility of heterotrimer formation between hMIF and *Ace*MIF monomers was investigated to analyze whether parasitic MIFs could interfere with hMIF trimerization [71]. The probability of such heterotrimer formation was excluded.

Despite all MIF homologs described in nematodes showing enzymatic activity, the activity is weaker in comparison to hMIF [75]. The enzymatic activity of MIF tautomerase affects the amino-acid residues Pro-2, Lys-33, Ile-65, Tyr-96, and Asn-98. For the oxidoreductase activity of MIF, the motif C–X–X–C is required [76,77]. Similar to hMIF, nematode homologs of *Ce*-MIF-1 possess tautomerase and oxidoreductase activity. The conserved proline residue at the *N*-terminal end and the C–X–X–C motif are necessary for the two activities. This was confirmed, for example, for *Bm*-MIF-1, *Wb*-MIF-1, and *Tci*-MIF-1 [60,62,65,73]. Oxidoreductase activity is not a characteristic enzymatic activity of MIF homologs, and the C–X–X–C motif is not present in all helminth orthologs [8]. Sharma et al. [65] showed that r*Wba*-MIF-1 exhibits oxidoreductase activity against insulin, thus suggesting that it is functionally active and similar to the native protein. Surprisingly, *Wba*-MIF-2 lacks the C–X–X–C motif, but significant oxidoreductase activity also was found in the insulin reduction assay. This is probably due to the presence of other vicinal cysteine residues. Homology modeling showed that of two of three cysteine residues (Cys58 and Cys95) are nearby (3.23 Å) in the tertiary structure with a pKa value of 9, indicating that they may play a role in the catalytic activity of disulfide oxidoreductase. Mutagenesis of these residues resulted in the lack of oxidoreductase activity in the insulin reduction assay, indicating that these two cysteines are crucial for the catalytic activity of *Wba*-MIF-2 [66,78].

It is worth mentioning that MIF’s ability to catalyze tautomerization of l-dopachrome methyl ester has contributed to the first study of native MIF orthologs in parasitic helminth species. MIF presence was detected in homogenate from L4 stage larvae of *T. spiralis*, as well as adults of *Trichuris muris* and *Brugia pahangi*, on the basis of dopachrome tautomerase activity. The activity was not detected in extracts from other helminth species tested: *Heligmosomoides polygyrus*, *Nippostrongylus brasiliensis*, *Hymenolepis diminuta*, *Schistosoma mansoni*, *S. japonicum*, *S. haematobium*, and the free-living nematode *C. elegans* [79].

In addition to their action as a host cytokine mimics, MIF homologs may play a role in the nematode physiology which relies on dopachrome tautomerase activity. The tautomerization of l-dopachrome is one of the steps of melanin biosynthesis and melanotic encapsulation, which is a key process in innate immunity to invading pathogens in a number of invertebrates [25]. Moreover, Nisbet et al. [73] suggested that the process of melanization may play a role in the protection of free-living *T. circumcincta* parasitic stages from UV exposure.

MIFs are expressed in various stages of nematode development. Pastrana et al. [60] showed that *Bm*-MIF-1 is produced in all developmental stages, with transcript levels in microfilariae and adults approximately twice as high as in L3 and L4 stages. Presence of the protein in the hypodermis, in the uterine lining, and on the surface of the muscle bundles was detected by immunolocalization techniques. This distribution pattern in the tissues of adult *O. ostertagi* [22], *T. circumcincta* [73], and *C. elegans* [56] has also been confirmed. nMIFs were present in all *T. circumcincta* and *O. ostertagii* stages; however, *Oos*-MIF-1 expression was higher in adults. On the contrary, *Tci*-MIF-1 levels appear to be higher in the egg and L3 larva [22,73]. In the case of parasites which infect the host through skin penetration, such as hookworms and threadworms, the highest level of nMIFs was noted in the infective stages. *Sra*-mif expression was higher in iL3 (infective third-stage larvae) than parasitic and free-living females [57], and *Ace*MIF was produced by L3 larvae (infective stage), but also by adult worms. It was not detected in eggs or newly hatched L1 larvae. The authors suggested that native *Ace*MIF is present only during those phases of the parasite’s life cycle that meet the host’s immune response, i.e., during migration within tissues and during attachment to the gut [71].

## 5. nMIFs and the Immune System of the Host

MIF activates cells by engaging its cell surface receptor CD74 [80]. A solid-phase binding assay showed *Ace*MIF also interacts with the hMIF receptor. Nevertheless, it was only partially effective in displacing hMIF from CD74. This suggests that hMIF and its homologs may bind to the receptor via different mechanisms. It is not entirely clear whether MIF homologs work as agonists, driving activation of downstream proinflammatory pathways, or as antagonists, engaging CD74 in a nonproductive or inhibitory manner [8,71]. Binding to the CD74 receptor was also confirmed for recombinant MIF homologs from *O. ostertagii* [22] and *S. ratti* [57]. Helminth secretion of MIF at the site of infection can also induce production of endogenous host MIF and lead to AP-1-mediated blocking of proinflammatory gene expression by binding of the transcription factor Jun activation domain-binding protein 1 (JAB1) [81]. Moreover, Wang et al. [72] showed that MIF from *H. contortus* is internalized by goat monocytes possibly inducing biological effects upon release from the endosome. Other studies proved that nMIFs are able to bind to human JAB1 molecule [62,69]. Whether this interaction leads to similar effects as endogenous MIF/JAB1 binding remains to be confirmed.

The first example of a parasite-derived molecule with significant homology to a host cytokine that functions to alter host cell behavior was described in *B. malayi* by Pastrana et al. [60]. To determine whether *Bm*-MIF had any direct effect on human peripheral blood monocytes/macrophages and to test the hypothesis that *Bm*-MIF could modify the activity of hMIF, a migration study was performed. Results of in vitro macrophage migration studies indicated that both hMIF and *Bm*-MIF showed chemoattractant activity and immobilized the cells, thereby inhibiting their random migration. This effect was confirmed via neutralizing anti-MIF antibodies which restored the migration of cells. Similarly to *Bm*-MIF, recombinant *T. spiralis* MIF (*Ts*MIF) inhibited the migration of human peripheral blood mononuclear cells (PBMCs) [70].

In a separate study, Zang et al. [62] expressed *Bm*-MIF-1 and *Bm*-MIF-2, as well as their site-directed mutants (*Bm*-MIF-1G and *Bm*-MIF-2G) with Pro-2 replaced with Gly. The results confirmed that both native MIFs can chemotactically mobilize macrophages in a similar way to hMIF. Interestingly, the mutant recombinant proteins, showed a 10-fold reduction in chemotactic activity for human monocytes, suggesting the crucial role of Pro-2. Another effect observed after *Bm*-MIF-1 and *Bm*-MIF-2 stimulation was the induction of Ca^2+^ influx in human monocytes and upregulation of TNF-α and IL-8 expression, which was about 10-fold lower for the mutant proteins. Other cytokine genes, IL-1β, IL-10, IL-6, IL-12(p40), IFN-γ, macrophage inhibitory cytokine-1 (MIC-1), monocyte chemoattractant protein-1 (MCP-1), and macrophage inflammatory protein-1α (MIP-1α), remained at the same level after stimulation. Treatment of human monocytes with r*Bm*-MIF also induced the release of endogenous hMIF in vitro.

Chronic helminth infections are usually associated with a tissue-protective Th2 type of immune response with alternative M2 activation of macrophages, in contrast to classical M1 phenotype, which is characterized by the expression of high levels of proinflammatory cytokines, high production of reactive nitrogen and oxygen radicals, promotion of Th1 response, and strong microbicidal and tumoricidal activity [82]. M2 macrophages are induced by IL-4 and IL-13 [8,83], which leads to surface expression of IL-4R and CD206 (mannose receptor), upregulation of arginase-1 (Arg-1), chitinase-3-like protein (also known as Ym1 or ECF-L), and Resistin-like molecule (RELMα), and downregulation of NO production [84]. M2 macrophages are induced in the early stage of the anti-helminth immune response and are responsible for many actions leading to elimination of the parasite. First, they release a number of factors facilitating the development of type 2 immunity and recruitment of effector cells; moreover, they participate in parasite killing. Secondly, they are involved in tissue repair and tissue remodeling. Lastly, they limit excessive inflammation through the release of immunomodulatory cytokines. For an in-depth review of these mechanisms, please refer to Coakley and Harris [84].

A study by Filbey et al. [85] showed that the murine host MIF molecule plays a critical role in macrophage polarization into the M2 phenotype during *H. polygyrus* infection. MIF-deficient BALB/c mice were unable to reduce worm burdens or egg output following a primary infection due to insufficient M2 polarization. Interestingly, MIF activity was not detected in *H. polygyrus* extracts [79]. Other studies proved that nematode MIF analogs may also induce alternative activation of macrophages.

Surgical implantation of adult *B. malayi* worms into the peritoneal cavity of mice induced leukocyte progression, including M2 cells, as well as increased neutrophils and eosinophils [61]. Furthermore, the authors indicated that intraperitoneal injection of r*Bm*-MIF-1 is sufficient for eosinophil recruitment and M2 activation by increasing Ym1/ECF-L expression in the absence of active filarial infection. Moreover, the results showed the significance of *Bm*-MIF-1 amino-terminal proline since the *Bm*-MIF-1G mutant failed to induce Ym1 transcription in macrophages or mediate eosinophil recruitment.

A separate study by Prieto-Lafuente et al. [63] analyzed the activity of two MIF homologs from the nematode *B. malayi* in comparison with mouse MIF and found that *Bm*-MIF-1 and *Bm*-MIF-2 promote IL-4-dependent alternative activation of functionally suppressive macrophages in vitro. In vivo administration of r*Bm*-MIF-1/2 in mice induced the expression of markers that are specific for alternative macrophage activation, such as RELM-α and Ym1. Interestingly, murine MIF did not demonstrate similar effects, in contrast to the study by Filbey et al. [85], where murine MIF was indispensable for M2 polarization and parasite clearance. The authors suggested that, in *B. malayi* infection, parasite-released *Bm*-MIF may be the first stimulus for macrophages to initiate alternative differentiation, with host IL-4 being essential to complete this process [63].

A recent study confirmed the observation that nematode MIF molecules are responsible for M2 polarization. Recombinant MIF molecule from a nematode *Thelazia callipaeda* (*Tcp*-MIF) stimulated M2 differentiation in human THP-1 macrophages via TLR-4-mediated activation of the PI3K/AKT signaling pathway [67].

The effects of nematode MIFs on other aspects of the immune response were analyzed in various studies. Younis et al. [57] found that MIF released by *S. ratti* iL3 and parasitic females binds to host immune cells and generates distinct antibody responses, indicating its possible involvement in local parasite–host interaction. r*Sra*-MIF was found to induce IL-10 but not TNF-α production by PBMCs, in the presence or absence of polymixin B (PMB), ruling out a lipopolysaccharide (LPS) effect. These results were in agreement with Park et al. [3], who reported that IL-10 and TGF-β levels in bronchoalveolar lavage fluid (BALF) were substantially higher after mouse treatment with recombinant *A. simplex* MIF (r*As*-MIF). In addition, r*As*-MIF enhanced TGF-β and IL-10 production in the spleen and mesenteric lymph nodes, but there was no influence on the levels of IFN-γ, IL-6, and IL-13 [68]. r*As*-MIF appears to ameliorate dextran sodium sulfate (DSS)-induced colitis, suggesting that this molecule may be useful as a treatment for inflammatory intestinal diseases, as further discussed in the next section.

A similar cytokine profile was observed by Wang et al. [72] using goat monocytes stimulated with recombinant *H. contortus* MIF-1 (r*HC*MIF-1). LPS-induced production of proinflammatory TNF-α, IL-1β, and IL-12p40 was downmodulated, while IL-10 and TGF-β secretion was increased in a dose-dependent manner. This suggests that r*HC*MIF-1 contributes to the induction of an anti-inflammatory environment favorable for worm survival. In addition, this study showed that r*HC*MIF-1 significantly reduced NO production by LPS-treated goat monocytes. Furthermore, the phagocytic capacity, which is an early and fundamental step for effective removal of pathogens, was decreased in a dose-dependent manner [72]. The results suggest that r*HC*MIF-1 induced the alternative activation of goat monocytes/macrophages.

The effects of nematode MIF on MHC-I and MHC-II molecules was analyzed in r*HC*MIF-treated goat monocytes [72]. r*HC*MIF-1 was capable of inhibiting MHC-II expression on monocytes in a dose-dependent pattern, and no changes in MHC-I expression were observed. MHC-II molecules are constitutively expressed on the surface of antigen-presenting cells (APCs), which enables them to present extracellular antigens and initiate an adaptive immune response. Perhaps, nMIFs affect the presentation of antigens by decreasing MHC-II expression. At the same time, the endogenous antigen presentation pathway is not hampered, as the major function of MHC-I is to present intracellular proteins to cytotoxic T lymphocytes [86,87]. However, more studies are necessary to confirm mechanisms involved in this phenomenon [72].

## 6. nMIFs in the Treatment of Autoimmune Diseases

There is a gradual increase in the prevalence of various immunological disorders in developed countries [88,89]. The link between parasites and allergies or autoimmune diseases has been described as the “hygiene hypothesis” [90,91]. This hypothesis suggests that lack of exposure to parasites in childhood suppresses an immature immune system, resulting in a higher frequency of allergic and immunological diseases: asthma, allergic diseases, RA, cardiovascular disease, multiple sclerosis, type 1 diabetes, and inflammatory bowel disease (IBD) [92,93,94,95,96]. Animal studies have shown that parasite homogenates or secretions can suppress the immune response in the host, suggesting that immunosuppressive activity is induced by the parasite [97]. Experimental studies in animal models have shown that infection with helminths such as *S. mansoni*, *H. diminuta*, and *T. spiralis* can ameliorate colitis [98,99,100]. Extracts from nematodes such as *Angiostrongylus catonensis*, *Oesophagostomum dentatum*, and the already mentioned *T. spiralis* modulate the inflammatory reaction in allergic asthma models [101,102,103].

Several papers have shown that nematode-derived MIF homologs have immunomodulatory potential that can be used to treat allergies and autoimmune diseases. Park et al. [3] conducted a study in which they demonstrated that recombinant type 2 MIF homolog r*As*-MIF reduces the ovalbumin (OVA)-induced allergic airway immune response in mice. Treatment with r*As*-MIF in conjunction with OVA/alum during the provocation period induced total inhibition of eosinophilia and goblet cell hyperplasia in the lung and profoundly impaired the progression of pulmonary hyperreactivity. r*As*-MIF significantly reduced Th2-related cytokines (IL-4, IL-5, and IL-13) in BALF and allergen-specific IgG2a in serum. Levels of regulatory cytokines IL-10 and TGF-β in BALF in the r*As*-MIF-treated group were substantially increased compared with the other groups. Additionally, CD4^+^CD25^+^Foxp3^+^ T cells (regulatory T cells) were recruited to the spleen and lungs of r*As*-MIF-treated mice, and this recruitment was suppressed by anti-r*As*-MIF antibody.

Cho et al. [68] also conducted a study using the same protein (r*As*-MIF) to test whether it has the potential to attenuate DSS-induced colitis in a mouse model. They showed that r*As*-MIF suppresses intestinal inflammation and the production of inflammatory cytokines such as IL-1β, IL-6, TNF-α, and IFN-γ by recruiting Treg cells through binding to TLR2. The r*As*-MIF was found to exert anti-inflammatory effects by inhibiting epithelial and crypt cell destruction. Increased secretion of IL-10 and TGF-β by splenocytes and mesenteric lymph node (MLN) cells was also observed. These results suggest that r*As*-MIF appears to attenuate DSS-induced colitis and may be useful as a therapeutic agent for IBD.

Similar studies have been conducted using recombinant *W. bancrofti*-MIF-2 (r*Wba*-MIF-2). Administration of r*Wba*-MIF-2 markedly reduced the disease activity index (DAI) in mice with DSS-induced colitis. No blood in the stool was observed in mice treated with r*Wba*-MIF-2, and the colon length was similar to the control with only minimal inflammation and histological changes. Proinflammatory cytokine genes (TNF-α, IFN-γ, IL-1β, IL-6, IL-17A, and NOS2) were silenced in colon tissue and peritoneal macrophages of r*Wba*-MIF-2-treated mice, suggesting that r*Wba*-MIF-2 is a potent immunoregulatory molecule that can revert the inflammatory stimulus. Importantly, there was a significant increase in the number of IL-10-producing Treg and B1 cells in the colon and peritoneal cavity of mice treated with r*Wba*-MIF-2. The results indicate that r*Wba*-MIF-2 treatment can alleviate the clinical signs of DSS-induced colitis in mice by suppressing the inflammatory response in the colon [99].

## 7. nMIFs as a Potential Therapeutic Target

Vaccines or drugs targeting nMIFs may have therapeutic potential by prevention of infection or by facilitating expulsion of the parasite from the infected individual [8]. Only isolated reports of vaccine trials using nMIFs can be found in the literature. r*Tci*-MIF-1 was one of the components of a multi-antigen vaccine against *T. circumcincta*, which was administered to sheep. Although the vaccine was effective, the protective potential of the particular component of the vaccine is difficult to assess [104]. In a separate study, hamster vaccination with r*Ace*MIF provided partial protection from ancylostomiasis [105].

Differences in the three-dimensional molecular structure of human and parasitic MIFs molecules allow the efficient design of selective inhibitors. The study conducted by Cho et al. [71] proved that hMIF inhibitor ISO-1 did not inhibit *Ace*MIF tautomerase or chemoattractant activities. The catalytic site plays an essential role in the immunomodulatory activity of mammalian and nematode MIFs. Targeting this molecular interaction site provides a viable mechanism for blocking host and/or parasite cytokines [8,62,106]. Inhibitors of hMIF have been developed using rational drug design [107,108]. Similarly, selective nMIF inhibitors can also be designed on the basis of known active site and substrate structures. De novo identification of inhibitors or modification of currently available compounds can also be performed in silico [8,105]. Screening libraries of bioactive compounds can be an effective strategy for repositioning FDA-approved drugs or discovering new pharmacophores. Cho et al. [105] presented the results of a high-throughput screening (HTS) of a library of clinically active small molecules targeting *Ace*MIF on the basis of inhibition of tautomerase activity and found promising compounds for therapeutic use. The effects of each inhibitor were studied in three assays: inhibition of catalytic activity, binding to the MIF receptor CD74, and *Ace*MIF-mediated monocyte migration. Six inhibitors were identified. These inhibitors may facilitate the study of *Ace*MIF function in *A. ceylanicum* biology and serve as leading compounds for new chemotherapeutic agents for the treatment of hookworm and perhaps other parasitic infections [8].

## 8. Conclusions

MIF homologs are released by number of nematodes to manipulate and modulate the host immune response. The discovery of the coding sequences of parasitic MIFs and the derivation of recombinant proteins have allowed a closer understanding of their structure and function. nMIFs have structural, catalytic, and cell-migration-inhibitory properties, and they show similar activity to mMIFs; hence, they may be used as immunomodulators. These results will contribute to elucidating the molecular basis of parasite–host interactions, which are essential for understanding the course of parasitic infections. Further characterization of nMIFs is expected to contribute significantly to the development of novel therapeutic strategies in parasitic, inflammatory, and autoimmune diseases.

## Figures and Tables

**Table 1:** Parasitic nematode MIFs (nMIFs) expressed as recombinant proteins. The table contains cDNA sequences of nMIFs expressed as recombinant proteins.

Order	Species	Accession Number	Acronym	References
Rhabditida: Onchocercidae	*Brugia malayi*	U88035.1	*Bm*-MIF *Bm*-MIF-1	[60,61,62,63]
	*Brugia malayi*	AY004865.1	*Bm*-MIF-2	[62,63]
	*Onchocerca volvulus*	AF384027.1	*Ov*MIF-1	[56,64]
	*Onchocerca volvulus*	AF384028.1	*Ov*MIF-2	[56,64]
	*Wuchereria bancrofti*	AF040629.1	*Wb*-MIF *Wb*-MIF-1	[60,65]
	*Wuchereria bancrofti*	KJ939449.1	*Wba*-MIF-2	[66]
Rhabditida: Thelaziidae	*Thelazia callipaeda*	No data	*T.ca*-MIF	[67]
Rhabditida: Anisakidae	*Anisakis simplex*	EF165010.1	*As*-MIF	[3,68,69]
Rhabditida: Stronyloididae	*Strongyloides ratti*	FJ026392.1	*Sra*-MIF	[57]
Trichinellida: Trichinellidae	*Trichinella spiralis*	AJ012740.1	*Ts*MIF	[70]
Strongylida: Ancylostomatidae	*Ancylostoma ceylanicum*	EF410151.1	*Ace*MIF	[71]
Strongylida: Haemonchidae	*Haemonchus contortus*	CB012470.1	*HC*MIF-1	[72]
	*Ostertagia ostertagii*	BQ457911	*Oos*-MIF-1.1	[22]
	*Teladorsagia circumcincta*	FN599526.1	*Tci*-MIF-1	[73]
